# Effects of Post-Processing Parameters on 3D-Printed Dental Appliances: A Review

**DOI:** 10.3390/polym16192795

**Published:** 2024-10-01

**Authors:** Mana Hassanpour, Poom Narongdej, Nicolas Alterman, Sara Moghtadernejad, Ehsan Barjasteh

**Affiliations:** 1 Department of Chemical Engineering, California State University Long Beach, Long Beach, CA 90840, USA; mana.hassanpour01@student.csulb.edu (M.H.); sara.moghtadernejad@csulb.edu (S.M.); 2 Department of Mechanical and Aerospace Engineering, California State University Long Beach, Long Beach, CA 90840, USA; poom.narongdej@csulb.edu (P.N.); nicolas.alterman@student.csulb.edu (N.A.)

**Keywords:** 3D printing, post-processing, mechanical properties, washing, post-curing, dental applications

## Abstract

In recent years, additive manufacturing (AM) has been recognized as a transformative force in the dental industry, with the ability to address escalating demand, expedite production timelines, and reduce labor-intensive processes. Despite the proliferation of three-dimensional printing technologies in dentistry, the absence of well-established post-processing protocols has posed formidable challenges. This comprehensive review paper underscores the critical importance of precision in post-processing techniques for ensuring the acquisition of vital properties, encompassing mechanical strength, biocompatibility, dimensional accuracy, durability, stability, and aesthetic refinement in 3D-printed dental devices. Given that digital light processing (DLP) is the predominant 3D printing technology in dentistry, the main post-processing techniques and effects discussed in this review primarily apply to DLP printing. The four sequential stages of post-processing support removal, washing, secondary polymerization, and surface treatments are systematically navigated, with each phase requiring meticulous evaluation and parameter determination to attain optimal outcomes. From the careful selection of support removal tools to the consideration of solvent choice, washing methodology, and post-curing parameters, this review provides a comprehensive guide for practitioners and researchers. Additionally, the customization of post-processing approaches to suit the distinct characteristics of different resin materials is highlighted. A comprehensive understanding of post-processing techniques is offered, setting the stage for informed decision-making and guiding future research endeavors in the realm of dental additive manufacturing.

## 1. Introduction

Due to the growing demand for dental appliances, additive manufacturing (AM), also known as three-dimensional (3D) printing, has emerged as a preferred alternative to traditional methods, effectively addressing the time-consuming and labor-intensive nature of conventional techniques. Advances in digital technology for material processing in dentistry have enabled more predictable and efficient outcomes in dental laboratory operations [1,2,3]. Since Charles Hull introduced 3D printing in 1986, the technology has seen significant evolution and widespread adoption across various industries [4]. The flexibility of 3D printing, made possible by computer-aided design (CAD) models, allows for the production of intricate shapes and surfaces that are difficult to achieve with traditional manufacturing methods [1,4]. Today, 3D printing materials come in various forms, including polymeric resins, composites, metals, ceramics, biomaterials, and even food-based materials [5]. This technology offers numerous advantages, such as reduced material waste, minimal post-processing, lower production costs, faster manufacturing times, reduced heat and noise during fabrication, decreased wear on finished products, and the ability to produce a single, complex part instead of multiple assembled components [1,2,3,6,7]. Utilizing CAD programs, 3D printing enables the creation of complex, customizable parts with precise control over internal structures, allowing for designs that improve strength-to-weight ratios [1,2,3,6]. Additionally, digital design files can be transferred electronically, making it easy to share and store without requiring physical space [3].

**Figure 1:**
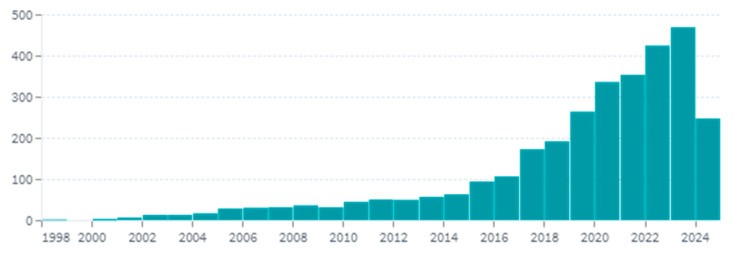
The trend of 3D printing publications in dentistry over time (www.lens.org).

Despite the surge in 3D printing technologies and related publications in dentistry over the past few decades (Figure 1), there is a notable absence of comprehensive reviews focused specifically on post-processing techniques for 3D-printed dental appliances. This gap in the literature poses challenges for practitioners and researchers who seek to optimize these techniques to enhance the properties of dental devices. The steady increase in publications underscores the growing interest and advancements in this field, yet it also highlights the need for a focused review on post-processing, a critical aspect that has not been adequately addressed. Therefore, this review aims to systematically analyze and discuss the various post-processing steps required for 3D-printed dental devices, offering valuable guidance for both current practice and future research by highlighting the significance of precision in post-processing to achieve optimal outcomes. Ultimately, a detailed review on this topic is essential to provide insights into effective post-processing methods, improving the mechanical strength, biocompatibility, and durability of 3D-printed dental products.

This review paper is organized into six main sections. Section 1 provides an introduction to the topic. Section 2 discusses 3D printing in dentistry, including the various fabrication techniques, materials used, and their required properties, as well as the overall workflow of 3D printing. Section 3 addresses the importance of post-processing, considering factors reported in previous studies. Section 4 outlines the specific post-processing steps, detailing support removal, cleaning, post-curing, and polishing techniques. Section 5 examines the problems, challenges, and future directions for dental 3D printing, focusing on current limitations and potential advancements. Finally, Section 6 provides concluding remarks, summarizing the key insights and emphasizing the significance of optimizing post-processing techniques in dental 3D printing.

## 2. 3D Printing in Dentistry

### 2.1. Classification of 3D Printing Techniques

3D printing technologies are categorized by forming processes like melting, sintering, and stereolithography, which use energy sources to either liquefy materials or induce bonding without liquefaction, as in sintering and stereolithography [1,8]. The American Society for Testing and Materials (ASTM) International classifies 3D printing into seven categories: VAT Photopolymerization (VP), Material Jetting (MJ), Binder Jetting (BJ), Material Extrusion (ME), Powder Bed Fusion (PBF), Sheet Lamination (SL), and Direct Energy Deposition (DED) [1,6].

In Material Extrusion (ME), fused deposition modeling (FDM) is popular due to its simplicity and low cost, involving thermoplastic filaments extruded through a nozzle. However, FDM lacks the precision of other methods, making it less suitable for high-accuracy applications like dental 3D printing [9]. MJ and BJ use droplets to form objects, with MJ relying on photosensitive resin solidified with UV light, while BJ jets a binder onto a powder bed. Although more precise than FDM, MJ and BJ are costlier and less common in commercial use. MJ is particularly useful in dental 3D printing for precision [1,9,10,11,12].

Powder Bed Fusion (PBF) uses lasers to fuse powdered materials, producing parts similar to bulk materials but mostly limited to thermoplastics in polymers. Direct Energy Deposition (DED) feeds material through a nozzle while melting it with a high-energy source, suitable for metals. Despite their precision and durability, PBF and DED are rarely used in dentistry due to issues like surface roughness and porosity [1,9,13].

Sheet Lamination (SL) bonds material layers, often with ultrasonic additive manufacturing, but is not widely used in dentistry, which demands small, precise parts [1]. VAT Photopolymerization (VP) uses photosensitive resins that solidify when exposed to light. These resins, mixed with additives, maintain stability and flexibility during the curing process. VP includes technologies like stereolithography (SLA), digital light processing (DLP), Daylight Polymer Printing (DPP), and Continuous Liquid Interface (CLIP) [1,6,10].

SLA, the first 3D printing process, uses CAD and Standard Tessellation Language (STL) files, which slice into 2D images projected onto a photosensitive resin. A laser traces the images, inducing polymerization layer by layer. The process prevents adhesion to the window using a polydimethylsiloxane (PDMS) coating [6,10,14]. DLP, similar to SLA, projects UV light onto the build platform using a digital micromirror device (DMD), with the resolution depending on the projector’s pixel size. DPP uses liquid crystal displays for resin curing with visible light, while CLIP employs an oxygen-permeable membrane to create “dead zones” that prevent unwanted polymerization [6,10,11,15].

In the realm of 3D printing, each device comes with its set of merits and demerits, necessitating careful evaluation for practical deployment in dental applications. Ideally, these processes should exhibit attributes such as swiftness, cost-effectiveness, high resolution, high surface quality, and excellent biocompatibility. FDM printers are acknowledged for their capacity to produce robust parts with commendable surface finishes, all while being recognized for their speed and affordability. However, achieving the requisite precision demanded by dental applications may entail opting for slower printing parameters and higher-cost alternatives. MJ options deliver impressive quality and expansive build areas, which translates into reduced printing durations. Nonetheless, these devices often bear higher costs and offer a limited selection of materials characterized by relatively lower mechanical properties. The sintering techniques are capable of generating precise and mechanically resilient components from a diverse range of materials. However, their operation necessitates extended printing times and an initial substantial investment. While printing with polymers considerably reduces costs, it often comes at the expense of mechanical properties, primarily stemming from less-than-optimal solidification. LOM, on the other hand, emerges as an economical and rapid method, best suited for larger components, rather than precision-critical applications [1].

Within the spectrum of dental 3D printing, VP techniques, particularly SLA and DLP, hold a prominent position [2,10,11,16,17,18]. SLA excels in producing high-precision parts with intricate designs and admirable surface finishes. However, its trace-curing approach extends total print times [1]. Consequently, DLP has garnered increasing favor within dentistry due to its full-layer illumination of photosensitive resins, facilitated by a “light pattern generator.” This methodology achieves comparable high resolution and quality while significantly enhancing printing speed and cost-efficiency relative to SLA [2,16]. Moreover, DLP exhibits reduced susceptibility to oxygen inhibition when compared to SLA, attributed to the location of the resin layer being polymerized, which remains consistently at the bottom of the vat, minimizing direct contact with ambient air [19].

### 2.2. Importance of 3D Printing in Dentistry

Over the past few decades, 3D printing technologies have emerged as pivotal tools in the realm of dentistry, offering compelling advantages, including cost reduction and the customization of devices tailored to individual patient needs [4]. Among the various 3D printing methods, VAT polymerization via SLA or DLP stands out as one of the oldest and most widely adopted techniques in dentistry. This prominence owes itself to its exceptional attributes, encompassing high accuracy, impeccable surface finish, capacity for producing intricate shapes with remarkable resolution, cost-effectiveness, single-unit fabrication capability, and expedited production compared to conventional methodologies [3,4,6].

**Figure 2:**
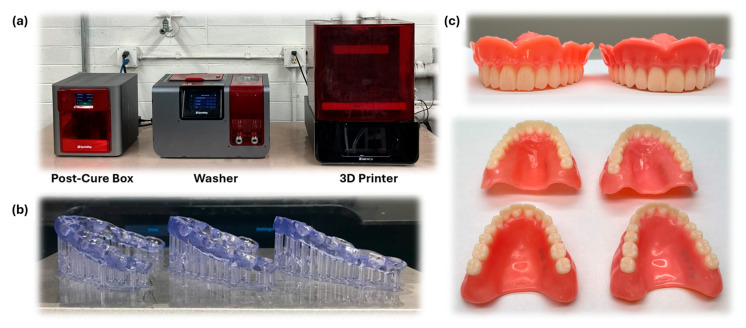
Applications of 3D printing in the dental industry. (**a**) 3D printing devices: printer, washer, and post-curing device. (**b**) Partially cured nightguards from the DLP printer. (**c**) Fully cured dentures fabricated via DLP printing apparatus.

In recent times, the range of dental devices manufactured via 3D printing has significantly expanded, encompassing a diverse array of applications (Figure 2). These include the creation of surgical guides, provisional crowns, dental splints, denture bases, implants, clear aligners, and mouthguards, to name a few [2,3,6,7,20,21,22,23]. The transformative impact of 3D printing extends across various dental specialties, including prosthodontics, orthodontics, endodontics, maxillofacial surgery, and oral surgical planning procedures [3,4,6,24]. This is chiefly attributed to the high accuracy and precision achieved through 3D printing, resulting in minimally invasive procedures, enhanced patient comfort, and superior aesthetic outcomes [3,4,24].

To ensure the successful application of these devices, they must meet specific clinical demands, which are guided by several general requirements that apply across all types of 3D-printed dental appliances [16,17].

3D-printed dental appliances must meet several critical requirements to ensure their suitability for clinical use. These general requirements include the following:

- Biocompatibility: the materials used must not elicit any adverse reactions when in contact with oral tissues [17];
- Mechanical strength: the appliances must be durable enough to withstand the forces exerted during mastication and other oral functions [17,19];
- Dimensional accuracy: precision in the dimensions is crucial to ensure a proper fit and function, particularly for items like crowns, bridges, and aligners [16,17];
- Surface quality: a smooth surface finish is necessary to prevent microbial colonization and enhance patient comfort [19].
- Durability and stability: the appliances should maintain their properties over time, even when exposed to the harsh conditions of the oral environment, including temperature changes, moisture, and mechanical wear [18,19].

By addressing these requirements, 3D-printed dental devices can achieve optimal clinical outcomes.

At the core of 3D printing’s success in dentistry is its ability to replicate complex oral structures with high fidelity. This is not merely a technological feat, but a phenomenon grounded in the logic of digital precision and material science. The virtual representation of dental models allows for meticulous planning and execution of treatment, ensuring that outcomes are predictable and reproducible [25,26].

For instance, in orthodontics, practitioners can visualize the final occlusion and plan the entire treatment sequence digitally. This logical progression from digital model to physical device streamlines the workflow, minimizes errors, enhances the overall treatment efficacy, and empowers the creation of anatomically precise structures tailored to each patient’s unique needs [3].

A prime example of the logical application of 3D printing is the use of surgical guides in implantology. These guides, designed using patient-specific data, ensure precise placement of implants. Research has demonstrated that the use of 3D-printed surgical guides significantly reduces surgical time and enhances postoperative outcomes, exemplifying the practical benefits of this technology [24,27]. Looking ahead, the integration of artificial intelligence with 3D printing promises to revolutionize dental care further. AI-driven design algorithms can optimize the fit and function of dental devices, while new materials with enhanced biocompatibility and durability are being developed. Ongoing research is exploring the potential for 3D printing in regenerative dentistry, where bio-printed tissues and scaffolds could one day replace damaged oral structures [28,29,30].

Beyond its clinical applications, 3D printing has the potential to foster improved communication between dental professionals and their patients [3,6]. Enhanced understanding of proposed treatments cultivates patient compliance, fosters mutual trust, and strengthens the practitioner–patient relationship. Additionally, 3D-printed devices can be fabricated using relatively inexpensive 3D printers within the orthodontist’s office, augmenting convenience and accessibility [3].

Moreover, the advantages of 3D printing extend to dental education, where dental students gain invaluable insights into the complexities of oral structures through 3D-printed models. These meticulously replicated models serve as educational aids, facilitating the assessment and tracking of students’ skills and progress [3,6,31].

A study conducted by Hegedus et al. [24] underscores the readiness of current and future generations of dentists to embrace digital technologies, demonstrating their seamless integration into the dental workflow.

### 2.3. Materials and Required Properties

The vat-polymerization process orchestrates the transformation of fluid materials into a solid state by converting aliphatic bonds into covalent bonds [2]. The performance of 3D-printed products, including their strength and reproducibility, is fundamentally dependent on the careful selection and composition of materials, as the physico-chemical properties of the printed parts are highly dependent on the materials used [4,32,33,34,35]. Consequently, comprehending the photochemical processes that underpin material response in the 3D printing process becomes pivotal in resin development, ensuring alignment with anticipated outcomes and desired properties [14].

In broad terms, 3D-printable resins comprise three principal constituents: (1) photosensitive monomers/oligomers housing reactive groups to construct polymeric networks, (2) photo-initiators, and (3) additives [2,36,37]. Monomers play a pivotal role in shaping the backbone of the cured part, exercising primary influence over its eventual physical and mechanical attributes. The choice of monomer must harmonize with the requisites of 3D printing, whilst also adhering to dentistry’s exacting standards, including high mechanical properties, chemical stability, biocompatibility, and the ability to confer desired aesthetic characteristics. Next, the photoinitiator assumes the vital role of initiating the polymerization process when subjected to specific light sources. Lastly, additives, including dyes or colorants, are judiciously introduced to fine-tune the ultimate appearance and control light penetration [36].

In the domain of denture manufacturing, acrylates derived from both natural and synthetic origins, exemplified by the macromolecule polymethyl methacrylate (PMMA), have emerged as the materials of choice. This preference stems from their attributes, including ease of processing, optical transparency, remarkable durability, robust wear resistance, tunability for achieving realistic appearances, high stability, suitability for sterilization, and biodegradability [5]. Additionally, other photosensitive thermosetting monomers employed in 3D printing for dentistry encompass urethane dimethacrylate (UDMA), Triethylene Glycol Dimethacrylate (TEGDMA), and Bisphenol A-Glycidyl Methacrylate (Bis-GMA). Nevertheless, due to their elevated molecular weight, viscosity, and limited availability, these materials are sparingly employed [2,38].

The study by Falahchai et al. [39] compared the mechanical properties of 3D-printed denture base materials with conventional heat-polymerizing denture base materials. It found that the 3D-printed materials had lower flexural strength, flexural modulus, and surface hardness but higher impact strength compared to conventional materials. This indicates that while 3D-printed materials offer certain advantages, such as higher impact strength, they may require further optimization in terms of flexural properties to match or exceed the performance of conventional materials.

### 2.4. Workflow of 3D Printing Technology in Dental Applications

**Figure 3:**
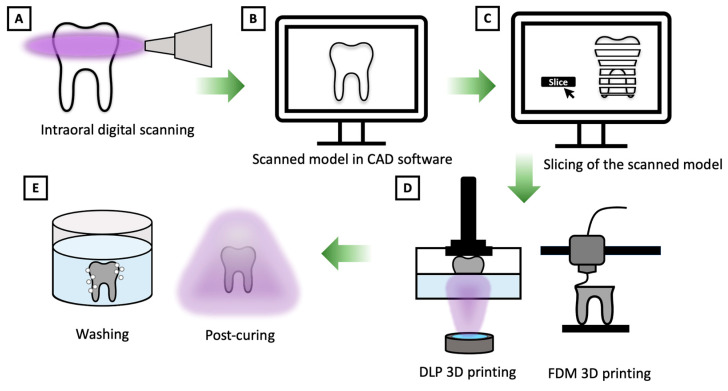
Summary of process procedures. (**A**) Intraoral digital scanning. (**B**) Scanned model in CAD software. (**C**) Cross-sectional slice of the scanned model. (**D**) 3D printing via (left) digital light processing (DLP) and (right) fused deposition modeling (FDM) techniques. (**E**) Post-processing treatments: (left) washing and (right) post-curing [1,3].

The comprehensive workflow of 3D printing technology in dental applications is illustrated in Figure 3. The process commences with the acquisition of a 3D model, which can be obtained through various intraoral digital scanning techniques such as computerized tomography (CT), cone beam computed tomography (CBCT), magnetic resonance imaging (MRI), or laser digitizing [3]. This scanned model can then undergo modifications using a CAD software such as 3Shape Dental System, CEREC SW, Excocad, etc. to align with the desired characteristics. Once the design reaches its final form, the software proceeds to convert the model into a series of cross-sectional slices encapsulated in an STL file format, which is subsequently dispatched to the 3D printer [1,3].

Subsequently, in the next phase, meticulous specification of the essential printing variables and parameters precedes the initiation of the printing process. The printer methodically fabricates the material in a layer-by-layer sequential fashion, with each newly printed layer seamlessly bonding to its predecessor. This iterative process continues until the entire part is successfully produced [1,3].

To achieve precise final designs and desired material properties, the meticulous determination of printing parameters assumes paramount importance. These critical parameters encompass build orientation, layer thickness, infill ratio, depth of cure, degree of polymerization, and the intensity of the light source. Build orientation, in particular, exerts a substantial influence on material properties, printing efficiency, final part accuracy, and the hygiene aspects of dental applications [4,32,40,41].

In the context of 3D printing efficiency, the choice of an appropriate build direction plays a pivotal role in optimizing the printing process. Specifically, selecting the right build direction minimizes the generation of support structures, thereby reducing the time required for subsequent surface finishing and polishing procedures [40]. For example, in the production of dental crowns, orienting the crown with the occlusal surface facing upwards can reduce the amount of support material needed, thereby decreasing post-processing time. Similarly, in creating surgical guides, aligning the flat surface parallel to the build platform minimizes support structures and enhances printing efficiency [40,42,43].

Moreover, precision and accuracy in 3D printing are intricately linked to the surface geometry and the extent of shrinkage between successive layers, both of which are significantly influenced by the chosen build direction. Consequently, the careful selection of an optimal build direction can enhance surface finish, mitigate shrinkage-related issues, and ultimately contribute to achieving higher levels of printing accuracy [32,40,41]. For instance, when printing dental aligners, a vertical build orientation can help maintain the integrity of the fine features, reducing layer shifting and shrinkage [44,45]. Given the anisotropic nature of 3D-printed materials, a profound understanding of how the mechanical properties of these materials is influenced by build orientation holds significant promise for enhancing the performance of fabricated devices [40]. Illustratively, a study conducted by Alharbi et al. [40] elucidated the relationship between build orientation and compressive strength, revealing that vertically printed components exhibited superior compressive strength when compared to their horizontally printed counterparts.

Furthermore, the work of Shim et al. [32] underscored the pivotal role of printing orientation in determining the hygiene aspects of dental prosthetics. This influence was attributed to surface characteristics, including roughness, hydrophilicity, and surface energy, all of which exert substantial influence on microbial adhesion to the printed components.

In summary, optimizing build direction not only enhances printing efficiency and accuracy but also plays a critical role in achieving the desired material properties that are essential for successful dental applications. By carefully considering the interplay between design parameters and material attributes, the performance and reliability of 3D-printed dental components can be significantly improved.

Despite the remarkable potential of 3D printing, certain inherent limitations exist within the technology. These limitations encompass relative fragility, susceptibility to environmental factors such as sunlight and heat, and the presence of uncured residuals post-printing [6]. Therefore, once the printed device experiences body temperature and intraoral humidity, the mechanical properties and original dimensions of printed components may undergo adverse alterations [29]. To mitigate these drawbacks, meticulous attention must be devoted to post-processing.

For 3D-printed constructs to be deemed fit for application, they generally require a series of post-processing steps to optimize their inherent properties [9]. These post-processing protocols are initiated after the detachment of the fabricated components from the printer’s platform. The workflow can be broadly classified into four pivotal stages: (1) mechanical excision of support structures, (2) solvent-based cleansing to eliminate residual resin, (3) additional removal of support materials, and (4) post-curing through controlled exposure to light and/or heat to attain the desired mechanical characteristics and biocompatibility [7].

Despite the importance of post-processing methods, available comprehensive reviews of these techniques are still limited. Therefore, this review paper aims to present an informative review of the recent 3D printing post-processing studies for dental applications. Herein, the importance of post-processing, its necessary considering factors, and its different stages are discussed.

## 3. Post-Processing

### 3.1. Importance of Post-Processing and Considering Factors

**Figure 4:**
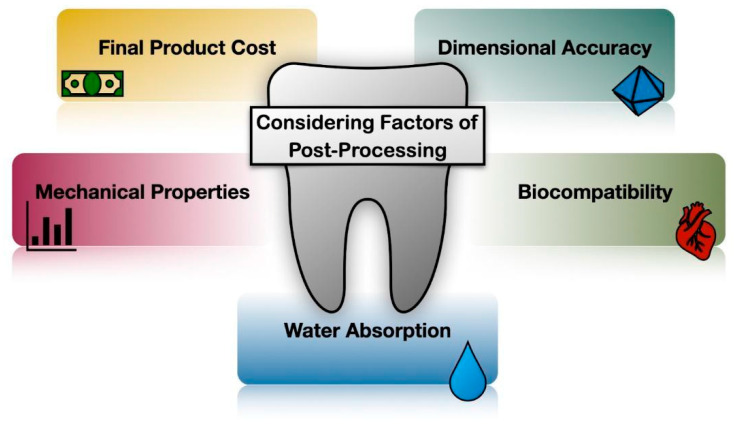
Considering factors of post-processing [18,19,44,46,47,48].

During post-processing, various transformations take place at the molecular level that significantly influence key attributes of 3D-printed models, including mechanical properties, biocompatibility, durability, dimensional accuracy, and aesthetic appeal [18,19,46]. The optimization of these properties is achievable through the careful adjustment of post-processing parameters, tailored to meet specific clinical requirements, such as the need for increased mechanical strength in crowns and bridges, or enhanced flexibility and durability in aligners. These specific clinical requirements are built upon the general demands outlined earlier, including biocompatibility, dimensional accuracy, and surface quality, but may vary depending on the particular application. For instance, surgical guides require a high level of precision and stability, while aligners need to balance flexibility with strength [44]. This involves selecting an appropriate method, determining the duration, and choosing the right solvent for the washing process, along with fine-tuning post-cure device settings including exposure time, temperature, and wavelength, which collectively contribute to notable enhancements in the final product [46,47,48], as summarized in Figure 4. Nevertheless, identifying optimal post-processing conditions is a complex task, as these conditions vary based on factors such as the type of resin used, the size and geometry of the print, and the specific end use [49]. The complexity is further heightened in models with internal geometry, even in cases as straightforward as a model with a hole [49].

However, the prediction of each factor’s impact on the final properties is hindered by several challenges. These include a limited understanding of polymerization behavior during printing, a lack of standardized protocols for post-processing [14,50], and a dearth of research, particularly in the dental sector, focusing on how various post-processing factors affect the final product’s characteristics [10]. Additionally, the disparity in resin types used across different studies poses a significant challenge in establishing a reliable comparison among the conducted tests and research findings. Despite these challenges, there is a general agreement in the field that the development of an optimal post-processing technique, particularly in terms of washing and post-cure factors, is critical for enhancing the final product’s accuracy, mechanical properties, surface quality, and biocompatibility [4].

Part of the post-processing procedure depends on labor, much of which is realized by hand. Hand processing can be time-consuming and subject to human errors [4]. This, in turn, has a significant impact on the economy of the process, which has been estimated to be about one-third of the production process cost of a 3D-printed part [51,52]. To overcome this shortcoming, an automation process should be further investigated and developed to reduce labor force requirements, time, and subsequent costs [53].

Post-processing shows little effect on the overall dimensional accuracy of 3D-printed specimens if the parts are fully cured during the printing process [10,11,20,54]. On the other hand, if the printed parts are not fully cured during printing, considerable shrinkage and shape distortion may be induced via the post-treatment [19]. Shrinkage occurs due to the reduction in volume as monomers are converted into a polymer network. This could be due to inhomogeneous curing during photochemical reactions or unstable temperatures during the printing process [16]. This can cause dimensional inaccuracies and potential warping of the printed parts. The deviation in the dimensional accuracy is critical in some applications, such as directly printed aligners, which leads to an undesired tooth movement [11].

Proper washing solution and washing time ensure the biocompatibility of the printed object by removing residual resin from the surface of the printed part [7,12]. However, over washing the samples may have detrimental effects on the surface quality and mechanical properties due to the surface absorption of the solvent, leading to plasticizing effects. Similarly, excessive post-curing may lead to depolymerization, brittleness, and inferior material properties [55].

To address issues related to post-processing, it is crucial to establish standardized protocols and develop comprehensive guidelines that account for various resin types and printing conditions. Implementing best practices for washing and post-curing can help mitigate the negative impacts on mechanical properties and dimensional accuracy. Additionally, employing advanced monitoring and control systems during post-processing can ensure consistent and optimal results, reducing variability and enhancing the overall quality of 3D-printed dental parts [56].

In addition, it has been found that proper post-processing procedures could reduce the staircase effect of layer-by-layer production, which is a poor surface finish commonly found in most 3D-printed parts [57]. Proper post-processing also improves biocompatibility, elastic and flexural strength, and stiffness compared to the green-state samples (i.e., before post-processing treatment) [4,16]. Flexural properties determine the material’s ability to resist deformation under applied intraoral forces. Dental appliances with low flexural modulus and strength tend to undergo repeating elastic deformation, leading to wear generation and detachment. Therefore, high flexural properties are needed in most dental appliances to prevent these problems [7].

However, the flexural properties of 3D-printed materials are generally low due to the combination of the reactivity of monomers and curing conditions, leading to a lower degree of double-bond conversion compared to typical conventionally processed resins. Moreover, weak interlayer bonding between printed layers is another cause for the lower mechanical performances [2]. Tartaglia et al. [20] pointed out that the mechanical properties of 3D-printed parts are generally anisotropic and highly sensitive to the post-cure and environmental conditions. On the other hand, Unkovskiy et al. [42] did not find any relation between the post-curing polymerization process and flexural properties.

Biocompatibility is one of the major material properties required in biomedical applications. One of the major obstacles found in photosynthetic resins is cytotoxicity due to the uncured monomers and residuals left on the part’s surface after printing. According to medical standards, the amount of surface residue is required to be lower than 18 wt%. This is because the remaining uncured monomers can act as a substrate promoting the growth of dental decay bacteria [7,12,36,58,59]. Especially in orthodontic applications, the presence of methacrylate monomers in directly printed aligner materials can be a source of genotoxicity, which can impact the DNA by producing reactive oxygen species [60].

Studies conducted by Tartaglia et al. [20] and Ahamed et al. [60] compared three commercially available dental resins, including Invisalign^®^, Dental LT^®^, and Accura 60^®^, in producing clear aligners. Even though Invisalign^®^ exhibited the lowest cytotoxicity, it could not be directly printed as the other two. Considering the ability to be directly printed, Dental LT^®^ was found to possess lower cytotoxicity than that of Accura 60^®^. The improvement in biocompatibility can be attained by eliminating the double bonds and obtaining a fully solid state of the resin, increasing the degree of conversion, and removing the excess liquid resin. These can be accomplished by implementing appropriate post-processing [59,61]. Based on Lin et al.’s [17] study on bifunctional dental monomers like BisEMA, UDMA, and TEGDMA, post-curing significantly impacts the degree of conversion. A degree of conversion below 50% indicates unreacted monomers, which could potentially leach out and cause cell death. However, these phenomena were not observed in cytotoxicity tests, suggesting that isopropanol washing and UV post-curing reduce monomer leaching by tightening polymer chains and enhancing the overall degree of conversion through non-uniform spatial conversion. In most 3D printing resins, toxicity measurement is usually performed once the parts are fully cured [17,49].

Water absorption is another important factor affected by the post-processing procedure, which has a negative impact on the biocompatibility and degradation rate of the dental 3D-printed parts, as well as other adverse effects, including dimensional changes, retention loss, and margin contour fracture of the dental crowns [62]. Typically, water uptake in resin materials occurs via diffusion, in which water penetrates empty spaces, such as micro-voids, or through molecular interactions. The molecular interaction process mainly depends on the resin polarity (i.e., the number of polar sites that are accessible for hydrogen bonding with water). These water–polymer chain interactions cause a reduction in the material’s strength, chemical degradation, and release of residual monomers. 3D-printed materials generally possess higher water solubility than conventional heat-cured materials because the 3D-printed parts are exposed to elevated temperatures for a shorter period of time. Therefore, the post-processing procedure is highly recommended in 3D printing fabrication [2].

Lastly, due to the uniqueness of properties and curing requirements of each photocurable resin, the post-processing factors should be specifically considered and determined to achieve the desired final attributes [4,6,7,14,55,57].

The critical and non-critical attributes impacted by post-processing input variables are summarized in Table 1.

### 3.2. Post-Processing Techniques

**Figure 5:**
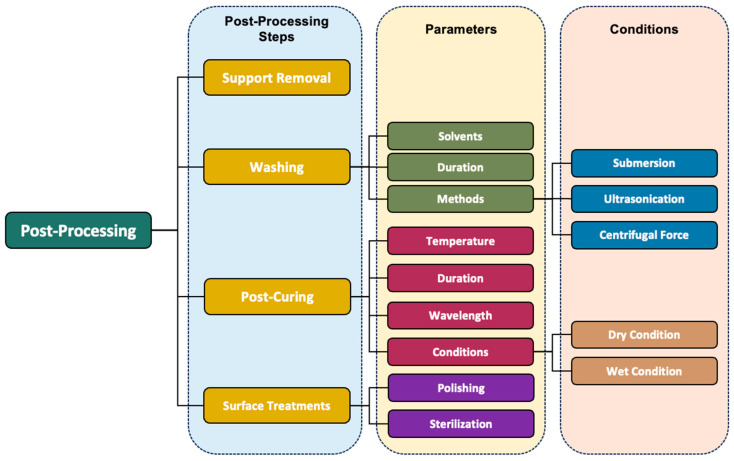
Summary of post-processing steps and techniques.

In general, post-processing can be divided into four steps as shown in Figure 5. These steps consist of (1) removal of the support structure, (2) cleaning and washing, (3) post-curing, and (4) polishing and surface treatment. It should be noted that the sequential steps listed in this figure are not fixed and can be adjusted for each specific resin. The details of each step will be thoroughly discussed in the following sections, and the summary of previously selected studies of various post-processing parameters in 3D-printed dental devices is shown in Table 2.

## 4. Post-Processing Steps

### 4.1. Removal of the Support Structure

After the printing process is completed, the printed parts need to be detached from the building platform. To avoid any breakages or uneven surfaces, this process is usually performed using a sharp and hardened knife, a spatula, a diamond disc, or an ultrasonic tip [9,10].

### 4.2. Cleaning/Washing

The washing process refers to the elimination of the excess uncured resin from the surface, holes, and ridges of the printed part by dipping it in a specific solvent. In this process, the solvent’s molecules gradually penetrate from the surface into the resin matrix and eliminate the unreacted monomers. This has a softening effect on the printed part and can alter its properties [73]. Washing guarantees the product’s biocompatibility and affects the final product’s mechanical properties, surface properties, and dimensions [14]. The mechanical properties of dental 3D-printed products should be able to withstand large biomechanical loads, such as chewing forces. The washing parameters should be carefully considered to enhance biocompatibility without deteriorating the part’s mechanical properties [7]. Despite the importance of the washing process mentioned, the available resources are still limited [10].

#### 4.2.1. Solvent Selection for Post-Processing of 3D-Printed Parts

When selecting an appropriate solvent for the post-processing wash of 3D-printed parts, the principle of ‘Like Dissolves Like’ is typically followed. However, a significant challenge arises due to the lack of detailed information about the resin ingredients. Manufacturers often keep the composition of their materials confidential, providing only safety data sheets with limited information. This lack of transparency leads to uncertainty and inconsistency in research and application methods [21].

Isopropyl alcohol (IPA) is the most widely used solvent in both industry and research, often recommended by resin manufacturers. Despite its widespread use, IPA presents several drawbacks, including high flammability, volatility, and potential health hazards such as dizziness, severe eye irritation, and respiratory issues [7,73]. Ethanol is another commonly recommended solvent, but it shares similar risks with IPA [73].

Several research studies aimed to investigate the effect of different types of washing solutions on the biocompatibility, surface properties, accuracy, and mechanical properties (e.g., flexural strength, flexural modulus, toughness, etc.) of the printed samples.

The effects of washing solution on the biocompatibility of the printed resins are determined by measuring the cytotoxicity and viability of the printed resins. With insufficient washing, the excess unreacted surface monomers cannot undergo sufficient polymerization during the post-curing procedure, as the existing oxygen acts as an inhibitor. Thus, the presence of unreacted monomers causes low biocompatibility of the printed part. In general, 3D-printed dental devices are found to possess superior biocompatibility after being washed with the proper solvent for an appropriate amount of time. Hwangbo et al. [7] pointed out that determining a proper washing duration is more critical than the type of solvent used to increase viability as well as biocompatibility. A similar observation is also reported in the study by Lambart et al. [73], in which they found an insignificant difference in the level of cytotoxicity in the samples washed with different types of solvents.

Various types of washing solutions are found to affect the surface characteristics of the print differently, depending on the molecular weight and the diffusion rate of the solvent. These surface characteristics (e.g., surface texture and quality) are important because they directly affect other properties like water absorption, discoloration, accuracy, and microbial adhesion of the dental printed parts [73]. Regarding surface roughness, Lambart et al. [73] revealed no significant variation after treatments with different types of washing solutions. They also reported no preference for solvent selection to minimize microbial adhesion. Snowwhite et al. [63] categorized the washing solutions into aggressive and non-aggressive groups. Among nine different solvents, they found that aggressive solvents resulted in a matte surface quality, while the non-aggressive solvents’ outcomes were glossy and clear. IPA showed aggressive effects on the printed parts as it not only washed the excess resins but also affected the structure of the printouts, resulting in a rough surface. Gonzalez et al. [36] achieved higher optical transparency for the ethanol-washed group in comparison with the acetone group on their printed resin material consisting of polyethylene glycol diacrylate (PEGDA), 1,6-hexanediol diacrylate (HDDA), and bisphenol A ethoxylate diacrylate (BEDA) with phosphine oxide-based compound (BAPO) as a photoinitiator.

Accuracy can also be directly affected by surface topography and texture [42]. Up to date, no solvent has yet been determined to yield a printed part with precisely defined dimensions. One study in this regard showed that a tripropylene glycol monomethyl ether (TPM)-washed printed dental model resin group had better accuracy in comparison to the group washed with commonly used isopropanol (IPA) solvent, although none of them resulted in accurately predefined dimensions [10].

The rate of diffusion-controlled penetration of various washing solutions into the polymer matrix has a tangible impact on the mechanical attributes of the final construct. In an investigation by Snowwhite et al. [63], the effects of nine different solvents on the flexural modulus and ultimate stress of 3D-printed parts were systematically evaluated, using a group cleaned with compressed air and wipes as a control. Within the scope of the resin used in their study, water-based solvents were identified as not only effective in removing residual material but also in enhancing both maximum stress and flexural modulus, approximating the control group’s metrics. Conversely, although isopropanol (IPA) effectively removed excess resin, it detrimentally affected the components’ structural integrity and flexural modulus. The utilization of food oil as a washing agent enhanced mechanical properties but left residual contaminants, as revealed through morphological analysis. The study presented inconclusive results for monomeric solvents. Polyethylene acrylate (PEA)-washed samples exhibited a considerable decline in flexural modulus, while trimethylolpropane triacrylate (TMPTA) and hexanediol diacrylate (HDDA) displayed increased modulus values. The authors hypothesized that the aromatic structure of PEA facilitated its more effective integration into the polymer matrix, while the higher viscosity of TMPTA deterred similar interactions. The structural similarity between HDDA and PEA made the disparate results puzzling. Moreover, the employment of monomers as solvents raises pertinent questions regarding biocompatibility, health implications, and safety and disposal procedures.

In a study by Bardelcik et al. [64], the influence of an array of wash treatment components on the tensile behavior of stereolithography (SLA)-printed polymethyl methacrylate (PMMA) resin components was examined. The unwashed samples exhibited the highest peak stress but registered the lowest elongation, thereby indicating reduced toughness. Conversely, specimens washed with varied solutions—including 60% and 90% isopropanol, 5% and 2.5% hydrogen peroxide, and a specialized detergent formulation comprising 0.3 wt% Sodium Caprylyl Sulfonate (Bioterge PAS-8S^®^), 0.2 wt% C9-C11 Alcohol Ethoxylate (Tomadol 91-6), citric acid/KOH as pH adjusters, and deionized water demonstrated improvements in both elongation and overall toughness, albeit at the cost of a reduced average peak stress. Specifically, the detergent-washed samples experienced a modest decline in average peak stress but manifested a significant enhancement in elongation, thereby elevating toughness levels. This phenomenon was attributed to the absorption of surfactants from the detergent into the polymer matrix, thereby augmenting the material’s ductility. Notably, the highest toughness registering a 90% improvement over the control group was observed in the samples treated with a blend of 5% hydrogen peroxide and the aforementioned detergent. In general, the groups treated with hydrogen peroxide showed an increase in elongation. On the other hand, the lowest average peak stress occurred in the group washed with a solution of isopropanol 60%, hydrogen peroxide 5%, and the detergent. Although there was a great increase in elongation, it could not offset the large drop in the average peak stress [64]. Similar results were observed in the group washed with the combination of isopropanol 90% and hydrogen peroxide 2.5%.

Hwangbo et al. [7] utilized two types of resins and corresponding 3D printing technologies: NextDent C&B (ND) resin via a DLP 3D printer (Vertex-Dental, Soesterberg, The Netherlands) and Formlabs Denture Teeth A2 (FL) resin through an SLA 3D printer (Form 3, Formlabs, Somerville, MA, USA). The study assessed the impact of isopropanol (IPA) and tripropylene glycol monomethyl ether (TPM) as washing solutions on the mechanical properties of various 3D-printed dental appliances. They found TPM to be a promising alternative to IPA due to its lower flammability, toxicity, and volatility, albeit at a higher cost and longer washing duration. Interestingly, the IPA-washed samples displayed markedly higher flexural strength, whereas flexural modulus remained consistent across both IPA- and TPM-washed groups. These findings align with those from Scherer et al. [65], who reported increased flexural strength in dental components washed with 91% and 99% IPA compared to those treated with bio-ethyl alcohol, TPM, and a water-based solution, RESINAWAY (RA). Mayer et al. [67] further corroborated the influence of washing solutions on flexural strength in 3D-printed fixed dental prosthesis materials, indicating the lowest flexural strength in acetone- and butyl glycol-washed groups and the highest in those cleaned using centrifugal force with Yellow Magic. Additionally, the highest degree of conversion, suggesting enhanced mechanical properties, was observed in groups washed with butyl glycol and IPA [2,21,66,67].

In a study by Lambart et al. [73], various washing solutions such as isopropanol (IPA), ethanol, EASY 3D Cleaner (EYC), Yellow Magic7 (YM7), and RESINAWAY (RA) were employed to cleanse DLP-printed FREEPRINT^®^ Splint 2.0 dental resin (Detax, Ettlingen, Germany). The investigation revealed that samples washed with ethanol and EYC exhibited the lowest flexural strength among the tested groups. This reduction in flexural strength was attributed to the smaller molecular weight of ethanol compared to the other solvents used in the study. Ethanol’s lower molecular weight allows for easier dissolution into the linear polymer chains, thereby disrupting the polymer matrix and consequently diminishing the material’s flexural strength [73].

In a comprehensive study, Mayer et al. [68] evaluated the impact of various washing solutions and methods on the fracture load and two-body wear of DLP 3D-printed three-unit fixed dental prostheses (FDPs) intended for long-term application. The study compared these findings to milled temporary polymethyl methacrylate (PMMA) FDPs. Three resins—Freeprint Temp (FPT), GC Temp PRINT (GCT), and Nextdent C&B MFH, along with milled [TEL]—were employed. The washing protocols involved the use of isopropanol (IPA) and Yellow Magic7 (YEL) in a 5 min ultrasonic bath and centrifugal force (CEN) at 1500 rpm for 5 min. The study concluded that both material type and washing method had a significant influence on fracture load and two-body wear. TEL-printed components registered the highest fracture loads, while FPT materials exhibited the lowest. IPA-washed samples generally demonstrated lower fracture loads compared to the YEL and CEN groups. When subjected to simulated masticatory cycles with specified vertical and horizontal loads, TEL-printed resins displayed lower resistance and higher material loss compared to FPT, irrespective of the washing technique used. The absence of filler fractions suggested that material composition could affect wear resistance. It was also posited that the impact of washing methods on wear resistance was more pronounced for filled resins than for unfilled variants. Interestingly, milled PMMA FDPs showed lower wear resistance than their 3D-printed counterparts across various tests, and the cleaning method had no significant impact on the wear resistance of FPT materials. Similar outcomes in terms of vertical material loss were noted for other printed resins like NMF and GCT [68].

These studies demonstrate the significance of proper washing solution selection and its influence on the final properties of the printed part. As previously stated, different studies may present varying results depending on the type of materials used and the selected test conditions. For example, the layer thickness of the printed parts, which is one of the printing stage adjustments, can affect the flexural strength of the printouts; the parts with a 50 µm layer thickness show higher flexural strength than those with 100 µm [73].

#### 4.2.2. Influence of Washing Time

Determining the optimal washing time is pivotal for achieving the best properties in 3D-printed parts. In their study, Jang et al. [69] examined the effects of washing 3D-printed objects with two different solvents: isopropyl alcohol (IPA) and Tetramethylsilane (TPM). They found that a 10 min wash with either solvent maximized the degree of conversion and minimized the presence of residual monomers, with no significant variation in flexural strength and surface roughness across different washing durations.

Hwangbo et al. [7] explored the influence of washing time on cell viability in methacrylate-based polymers (including urethane dimethacrylate, propylidynetrimethyl trimethacrylate, and phosphine oxide), also using IPA and TPM. The presence of uncured monomers in these resins can adversely affect cell viability due to cytotoxic and genotoxic effects. Their findings indicated that extending the washing time up to 90 min significantly enhanced cell viability, likely due to the more effective removal of unreacted monomers from the surfaces of the printed objects. Additionally, the use of ultrasonic cleaners was shown to further improve cell viability. A key observation was that a minimum of 60 min of washing was required to achieve 60% cell viability, with marginal benefits observed beyond this duration. While increased washing time did not notably affect the flexural strength of the printed parts, a decrease in flexural modulus was observed, possibly due to the reduction in molecular weight of the resin over prolonged washing periods. This reduction might be responsible for the material’s increased capacity to bear loads during elastic deformation. Hwangbo et al. [7] thus recommended cautious consideration of washing duration, especially for dental prosthetics, suggesting that excessively long washing times be avoided.

Building upon these insights, Scherer et al. [65] observed that increasing the total rinsing time enhanced the flexural strength of DLP-printed specimens. Their study encompassed four distinct rinsing durations (5, 6, 7, and 8 min) and employed three different solutions (IPA-91, IPA-99, and TPM), revealing a positive correlation between rinsing time and flexural strength.

Xu et al. [70] provided a nuanced perspective, noting that a 5 min wash in IPA, supplemented with ultrasonication, effectively increased cell viability. However, they found no additional benefits to cell viability with washing times extending beyond this period. They maintained that flexural strength and surface morphology were consistent for wash durations ranging from 5 to 60 min. Notably, a decline in flexural strength and the emergence of surface cracks were observed when washing exceeded 12 h. This deterioration is attributed to solvent molecules penetrating the surface, causing expansion, stress, osmotic pressure, and relaxation of the polymer networks.

Nowacki et al. [71] further elaborated on these effects, focusing on the impact of washing duration on 3D-printed specimens immersed in propanol solvent. Their findings indicated that insufficient washing led to increased surface roughness. Conversely, extended washing durations adversely affected tensile strength, likely due to the formation of voids that resulted in heightened local stress concentration. Interestingly, they noted that flexural strength remained unaffected by variations in washing duration.

Gonzales et al. [36] introduced an innovative approach, utilizing ultrasonication to reduce cytotoxicity. They demonstrated that 3D-printed samples (composed of polyethylene glycol diacrylate (PEGDA), 1,6-hexanediol diacrylate (HDDA), and bisphenol A ethoxylate diacrylate (BEDA)) exhibited enhanced cell proliferation when sonicated for 5 min compared to those immersed in ethanol or acetone for 2 h. Remarkably, their study showed no presence of unreacted compounds post-washing, suggesting that potentially toxic substances were effectively extracted during sonication.

Oh et al. [72] examined how washing temperature and duration affect the mechanical and biological properties of 3D-printed resin. Their findings revealed that raising the washing solution temperature significantly enhanced the degree of conversion rate and cell viability. However, higher temperatures and longer washing times negatively impacted flexural strength and microhardness. Consequently, they suggested that selecting an appropriate washing temperature and time is crucial for optimizing biocompatibility while minimizing adverse effects on mechanical properties.

#### 4.2.3. Influence of Washing Method

The method of washing plays a crucial role in removing excess resin from the surface of 3D-printed parts, impacting their final properties. Lambart et al. [73] underscore the significance of not only the choice of solvent and duration of washing but also the washing technique itself. They point out that different approaches, such as utilizing sonicators, applying centrifugal force, or employing immersion baths, can lead to distinct outcomes in the final product characteristics. Jin et al. [74] reported that ultrasonic bath yielded a better outcome on the elution of residual monomers than that of a rotary washer. Moreover, they highlighted that extending the washing duration could further reduce the mechanical properties and cytotoxicity of 3D-printed parts.

Delving deeper, Virinthorn et al. [75] explored the implications of varying sonication power levels (0, 20% [22W], and 40% [44W]) and immersion times (5, 15, and 30 min) on the removal of poly (vinyl alcohol) (PVA) binder from Poly (lactic-co-glycolic acid) (PLGA) dental scaffolds. Their study, conducted with inkjet printing technology, used a solvent mix of acetone and ethanol, offering a combination of polar and nonpolar interactions. They observed that both sonication and prolonged immersion led to surface unevenness in the scaffolds. Interestingly, while sonication did not affect scaffold stiffness, it did facilitate the reduction in PVA content with increased sonication power and washing duration.

Additionally, a recent study by Vara et al. [46] assessed the impact of washing methods specifically for SLA- and DLP-printed parts. It compared handwashing, ultrasonic cleaning, and a combination of both. The study found no significant difference in dimensional accuracy between these techniques, but it highlighted the critical importance of thorough cleaning to remove uncured resin effectively. This finding underscores the necessity of meticulous washing protocols to ensure the optimal final properties of the 3D-printed parts.

However, it is noteworthy that research focusing on the effects of washing methods, particularly for stereolithography (SLA) and digital light processing (DLP) printing techniques, remains relatively limited. This gap highlights an area for further investigation to fully understand the impact of washing techniques on the surface quality and mechanical properties of parts produced via these printing methods.

### 4.3. Post-Polymerization (Secondary Curing Steps)

#### 4.3.1. Importance of Post-Curing

Post-curing, also referred to as post-polymerization or a secondary curing step, is a crucial process in resin 3D printing, significantly impacting the final product’s properties [9,10]. During initial polymerization, not all monomers are fully cured due to surface oxygen inhibiting the reaction [9,38,66]. This leaves the printed parts soft and bendable, with suboptimal mechanical properties, biocompatibility, and stability [66]. This process increases the degree of conversion (DC), mechanical strength, thermal stability, and chemical resistance of the printed parts. Initially, 3D-printed models exhibit a low DC due to the kinetics of the polymerization reaction. At the start, monomers are highly mobile, allowing efficient diffusion and promoting rapid polymerization until the gel point is reached, where polymer chains crosslink to form a three-dimensional network. After the gel point, monomer mobility significantly decreases, limiting their diffusion and reaction, leading to incomplete conversion and a lower overall DC. The post-curing process provides additional energy to drive further polymerization, enhancing the crosslinking of the polymer network and increasing the DC [96].

The degree of crosslinking is a crucial factor that determines the material’s rigidity and resistance to deformation under stress [96]. The thermal properties of the polymer, such as the glass transition temperature (Tg), are affected by the extent of crosslinking achieved during post-curing. A study by Cingesar et al. [97] demonstrates that Differential Scanning Calorimetry (DSC) tests show that post-curing reduces curing enthalpy and shifts the glass transition temperature to higher values, suggesting increased crosslinking of polymer chains. A higher degree of crosslinking generally results in an increased Tg, indicating improved thermal stability. Additionally, the mechanical properties, including tensile strength, flexural strength, and hardness, are significantly enhanced through optimized post-curing, as the polymer network becomes more tightly interconnected [98,99,100].

Typical post-curing methods include UV exposure in a UV chamber, heat treatment, and microwave processing. These techniques aim to achieve complete polymerization of residual monomers, forming polymer chains and enhancing crosslinking. This process is especially vital in denture 3D-printed devices, which have direct, prolonged contact with both hard (enamel, dentin, and cementum) and soft (pulp) oral tissues [2,4,9,10,38,41]. Post-curing not only improves mechanical properties and biocompatibility but also enhances dimensional accuracy [41]. In this regard, Mendes-Felipe et al. [33] found that the thermal properties of post-cured samples are similar regardless of whether heat or UV light is used, indicating no significant difference between heat or UV light post-curing. However, UV post-curing results in a more homogeneously high crosslinked material with superior mechanical properties.

Usually, post-curing conditions are controlled via temperature, duration, and post-curing methods, considering the predefined desired properties of the final product [49,66]. Factors such as pigmentation, material stability, composition, and the size and geometry of printed parts are also considered [2,66]. Additionally, the selection of post-curing equipment significantly influences the final properties of the printed models [101,102], a factor that is particularly critical for large or intricate designs. These items require uniform light exposure, which can be facilitated by using a turntable in the post-cure chamber, ensuring consistent curing throughout the piece [2,66]. In this regard, Bayarsaikhan et al. [102] found that the flexural properties of 3D-printed crown and bridge resins showed no significant differences when post-cured using various devices. However, considerable variations were observed in the degree of conversion, Vicker’s hardness, and cell viability. Lassila et al. [103] further evaluated the effects of various post-printing devices on the mechanical and optical properties of 3D-printed dental resins. Their study found that post-curing in a Form Cure curing unit yielded the highest flexural strength and lowest wear depth compared to other devices such as the Visio Beta Vacuum, Ivoclar Targis, and heat curing. Hydrothermal accelerated aging significantly reduced flexural strength across all groups, highlighting the need to consider environmental factors in post-processing. While the optical properties, including surface gloss and translucency, were largely unaffected by post-printing conditions, the polishing process had a significant impact on gloss values. SEM and EDS analyses confirmed that the micro-structure and elemental composition remained consistent, indicating that post-curing primarily affects mechanical attributes.

Obtaining an optimal condition for post-curing is essential, as over-curing may cause health issues by making the printed parts brittle and weak [49]. Monzon et al. [85] mentioned anisotropy as an issue in DLP printing technology that can be eliminated by a proper post-curing procedure. However, they stated that the effectiveness of this improvement could be affected by the presence of additional pigments. Cortés et al. [95] observed improved results in Young’s modulus and three-point bending strength on their DLP-printed developed carbon nanotube (CNT)-reinforced composites after performing post-cure heat treatment compared to the group with no post-cure. Perea-Lowery et al. [2] noticed a significant influence on the flexural strength of the 3D-printed denture base resins after post-curing. They performed tests for two groups of specimens, one in a dry condition and the other after 30 days of storage of the specimens in water. Both groups presented higher flexural strength after post-cure, but flexural strength and elastic modulus were significantly lessened after water storage. Riccio et al. [21] investigated the effect of post-curing on the mechanical properties of SLA printed on various commercial Formlab resins. They observed an improvement in tensile properties that could be obtained when the tests were performed after 24 h of post-curing the specimens. Although post-curing could increase both rigidity and resistance to tensile breakage of the printed parts, the final properties of the parts with either increased plastic elongation, pseudo-brittle, or entirely brittle specimens were mainly governed by the resin type and composition.

Brittleness is generally the result of shrinkage and plastic deformation, which are some of the SLA manufacturing limitations causing residual stress in the printed parts [4,21]. This can be drastically mitigated by post-curing [4]. Kim et al. [90] evaluated the effect of post-curing as well as the printing orientation on the shrinkage of the samples printed via DLP using their developed photocurable resin, a methacrylate PDMS-macromer consisting of 2,4,6-TrimethylbenzoyldiPhenylphosphinate, known as commercial TPO-L photoinitiator. Since they did not use any solvent or additives in their synthesized resin, their printed parts showed less shrinkage compared to commercial SLA or DLP resins. Solvent evaporation and additive expansion/shrinkage can be major factors contributing to volume change. Furthermore, the size of the samples is another factor affecting the shrinkage rate. Larger parts may shrink less due to being more exposed to light and requiring more time for printing, which implies more monomer conversion during the printing time and less polymerization during the post-curing. Among X-, Y-, and Z-orientation printed parts, Z-orientation printouts in all sizes had the lowest shrinkage due to better polymerization in each layer. They concluded that greater exposure time indicates better polymerization and a lower degree of additional polymerization during post-curing exposure time. In general, it can be seen how important it is for dentists and dental technicians to have a good insight into how varied post-curing conditions might affect the final attributes of dental printed devices [41].

#### 4.3.2. Effects of Post-Curing Temperature

Several studies show that increasing the post-curing temperature alters the chemical and mechanical properties, biocompatibility, and degree of conversion of the 3D-printed dental resins in addition to affecting the final shape and form of the printed parts [2,9,76,77,78,104]. Higher temperatures lead to a decrease in viscosity, increasing the collision of free radicals and providing more energy to initiate reactions for crosslinking formation [2,79]. However, it is essential to avoid excessive temperature levels that could cause material deterioration. For instance, polymethyl methacrylate (PMMA), one of the commonly used materials in the dental 3D printing field, cannot tolerate temperatures above 125 °C and starts to depolymerize, producing a methyl methacrylate monomer (MMA), with 90% of the polymer depolymerizing by 450 °C [5]. To prevent dimensional changes in 3D-printed denture base parts, Katheng et al. [79] recommend keeping the temperature at 66 °C or below. Shrinkage is another important factor that increases as the temperature rises which may affect the dimensional accuracy of the 3D-printed dental resins [80]. Hague et. al. [81] highlighted the importance of appropriate temperature control as too much temperature contributes to accelerating the aging process and other adverse impacts.

Alsandi et al. [11] investigated the effects of additional heat on the post-curing process and its impact on the mechanical properties of 3D-printed samples made from urethane dimethacrylate (UDMA). They found that combining light-curing with heat curing at 110 °C significantly improved the ultimate tensile strength compared to samples cured with light alone. This was due to the improvement in the polymerization process and enhanced interpenetrating polymer network at the interfaces between layers with the aid of additional heat. Moreover, heat treatment was also found to improve internal adaptation as well as the degree of conversion of UDMA material on the surface of specimens. The study suggested that the combination of light- and heat curing could be an alternative to achieve higher and more suitable properties for bioapplication 3D printing materials [11].

Jindal et al. [66] performed tests on Dental LT clear resin, a type of Class II, long-term biocompatible resin, to evaluate different groups of the printed dental aligners’ compressive resistance and compressive modulus. These aligners were cured at various temperatures and durations in an automated post-cure chamber. The study found that the aligners post-cured at higher temperatures demonstrated higher compression strength with the same post-cure durations. Compression strength testing was important as the printed aligners should be able to withstand the average human jaw’s bite force, which is typically between 50 and 700 N (on average 400 N). They exposed the printed aligners to a load of 1000 N and found that the uncured group of aligners could only resist 380 N with considerable deformation. Whereas the aligners that were post-cured at 80 °C showed significant improvements. They observed the aligners post-cured for 15 and 20 min at 80 °C exhibited higher compressive strength along with elastic manners, while those post-cured for 5 min at 80 °C showed plastic deformation. Similar results were observed for post-curing at 60 °C and 40 °C, demonstrating parts with sufficient strength for withstanding the average human jaw chewing process (400 N). Therefore, the researchers concluded that for the same post-cure duration, post-curing at higher temperatures results in higher compressive strength. The compression modulus also improved with increasing post-cure temperature and time. In terms of biocompatibility, they realized that when employing a Blue UV-A 315–400 nm or UV-Blue 400–550 nm source, post-curing for 10 min was required. Nevertheless, they specified that for the type of resin employed in their tests, 3D-printed dental aligners with better mechanical attributes were obtained with longer post-curing durations of 15–20 min at temperatures ranging from 40 °C to 80 °C, when a 405 nm light source was applied. They stated that by adjusting the post-curing conditions, personalized aligners could be manufactured based on each patient’s specific needs.

Bagis et al. [82] developed a linear equation with a slope of 0.15 representing how a rising post-curing temperature enhances the degree of conversion. The temperature was found to be twelve-times more effective than the post-curing interval. Greater monomer conversions were achieved for parts post-cured between 3 and 7 min compared to shorter post-curing times (30 s and 1 min) and samples without any post-cure. Furthermore, for the commercial resin composite, they found higher degrees of conversions were achieved at 100 °C to 125 °C, making intraoral post-curing impractical [82].

Katheng, et al. [79] conducted experiments on a methacrylate-based photopolymer resin (Clear resin, Formlabs) printed via the SLA technique to investigate the effect of post-curing duration and temperature on accuracy and the degree of conversion of unreacted monomers. The printed parts were washed with IPA for 15 min and then grouped for post-curing at three different temperatures: 40 °C, 60 °C, and 80 °C, with curing times of 15 and 30 min. They found better accuracy, in terms of both the fit and tissue accuracy, was achieved at lower temperatures. As the temperature increased the gap size also increased, leading to lower fitting accuracy. Moreover, dimensional changes occurred at higher temperatures, affecting tissue accuracy. They indicated that higher temperatures could cause distortion or shrinkage, depending on the type of materials and the geometry of the printed dentures.

Regarding the degree of conversion, post-polymerization at 60 °C for 30 min had the best result, while the least favorable results belonged to the group post-cured for 30 min at 40 °C. Although the degree of conversion showed better results at 60 °C, it decreased when the temperature was elevated to 80 °C. The researchers believed this reduction was due to the presence of oxygen clinging to free radicals at this temperature, acting as an inhibitor. In addition, high temperatures could negatively affect the aging process and cause shrinkage of the printed parts. Based on their findings, the researchers reported that the optimized post-curing conditions were 15 min of curing at 40 °C, which favored both accuracy and polymerization efficacy terms [79].

Miedzinska et al. [83] conducted compression tests at different strain rates on SLA-printed parts made from Durable Resin from Formlabs. They found that higher mechanical properties were achieved at higher post-curing temperatures in shorter post-curing times for all test conditions. Significant changes in mechanical properties for static and dynamic test conditions were observable when the parts were cured for at least 5 min, but no considerable changes were noticed for 30 and 60 min of post-curing. For SHPB (Split Hopkinson Pressure Bar) tests, curing time did not noticeably affect the strengthening process [83].

#### 4.3.3. Effects of Post-Curing Duration

Post-curing time for each material may differ from one another due to variations in wall thickness, internal structure, pigmentation, and geometrical configuration. These factors can lead to certain areas within the printed part being blocked from direct UV light exposure, resulting in non-uniform curing [49]. Therefore, it is necessary to have an appropriate post-curing duration to obtain optimal color stability and mechanical properties for 3D-printed samples [105].

The study by Kirby et al. [96] demonstrated a statistically significant improvement in the degree of conversion with longer post-curing times. Specifically, extending the post-curing time from 15 min to 45 min increased the degree of conversion, with the Phrozen Cure V2 achieving the highest mean degree of conversion at 69.6% after 45 min.

Kim et al. [41] reported that hardness, degree of conversion, and biocompatibility could be enhanced with prolonged post-curing duration. An enhancement of post-curing on monomer conversion, printing accuracy, and surface quality was also found in various studies [71,82,83,86,87]. While other properties were found to be enhanced with increasing post-curing duration, mechanical properties including tensile, flexural, and hardness were found to degrade if the post-curing duration was poorly chosen [71,85]. Furthermore, Nowacki et al. [71] pointed out that longer post-curing could potentially affect the dimensional accuracy due to the shrinkage of resin during crosslinking.

Scherer et al. [91] investigated the effects of various post-curing durations (ranging from 25 to 45 min) under wet (i.e., in water and glycerin) and dry conditions on fracture resistance and flexural strength. They found that the optimal result was obtained from the dry post-curing condition for 25 min. Furthermore, a study by Lowery et al. [2] highlighted the relationship between post-curing time and temperature, suggesting that a longer post-curing at a low temperature could yield comparable outcomes to shorter post-curing at a higher temperature.

In terms of accuracy, Katheng et al. [79] investigated the effects of post-curing time for 15 and 30 min at three different temperatures (i.e., 40 °C, 60 °C, and 80 °C) on the accuracy of the dental dentures. They concluded that the accuracy was less likely to be influenced by post-curing duration.

According to a study by Soto-Montero et al. [48], fine adjustment of post-curing times is crucial to achieve adequate mechanical properties without compromising color stability. For most evaluated resins, a post-curing time of 5–10 min resulted in acceptable flexural strength and flexural modulus with minimal color changes. Additionally, they specified that Knoop microhardness (KHN) was significantly influenced by post-curing time and depth, generally enhancing with longer post-curing times, particularly at deeper levels. The highest KHN values were recorded at the superficial measurement depth (50 µm), decreasing at deeper levels (2–4 mm), but increasing again at 5 mm depth. The study emphasizes the material-dependent nature of post-curing effects, advising that each resin’s specific post-curing time should be evaluated to balance esthetics and mechanical performance.

A study by Aati et al. [106] further reinforces these findings, demonstrating that extended post-curing durations significantly enhance mechanical properties such as flexural strength and modulus, and Vickers hardness. Additionally, based on their findings, longer post-curing times reduce cytotoxicity, ensuring the biocompatibility of the final product. This study also highlights the importance of post-curing in reducing water sorption and solubility, contributing to the long-term stability of dental appliances.

#### 4.3.4. Effects of Wavelength/UV Intensity of the Cure-Box

The effects of wavelength and UV intensity are considered important factors in achieving optimal properties and determining the effectiveness of post-curing procedures [89]. Raymus et al. [84] investigated the influences of different post-curing wavelengths and durations on fracture load values of various commercial 3D printing resins. Three post-curing methods were utilized in this study, consisting of (a) LED light with a wavelength of 380–510 nm for 6 min, (b) 4000 flashes with a wavelength of 300–700 nm in a nitrogen atmosphere, and (c) a UV light with a wavelength of 315–550 nm for 30 min. They found that the fracture load values from each method varied and were highly dependent on the types of resins. Even though the post-curing techniques are specific to each product, this study emphasized the importance of the correct post-curing strategy to ensure the properties of 3D printing resin materials [84].

Another study by Zachary Zguris [88] also investigated the effects of three different UV wavelengths (i.e., 365 nm, 385 nm, and 405 nm) on different FormLab resins including castable, standard, and tough resins, and concluded that the optimal tensile modulus and strength could be obtained with a 405 nm light source. Therefore, the 405 nm wavelength is one of the most commonly selected light sources in commercial products. During UV post-curing, the energy from the light source facilitates the formation of additional covalent bonds between polymer chains, resulting in a more robust network structure.

Wu et al. [16] reported that the UV intensity had no significant effect on the degree of conversion. They explain this is because the UV intensity dominates the rate of secondary curing, but it does not contribute much to the degree of conversion gradient, which determines the number of unreacted monomers. With the increase in the UV intensity, the amplitude of curvature was not significantly increased. Only the time required to reach the amplitude of curvature was shortened [16].

#### 4.3.5. Effects of Post-Curing Conditions

Several research studies have evaluated the effect of post-curing conditions on the attributes of printed components. Scherer et al. [91] conducted a study on NextDent C&B MFH N1 resin printed parts under three different post-curing conditions: (1) dry condition (simply placed in a cure box), (2) immersed in water, and (3) submerged in glycerin. Water and glycerin act as oxygen inhibitors during post-curing. The fracture resistance and flexural strength of the samples were tested after each of these post-curing conditions. The best results were achieved for the dry group, followed by the water-soaked group, which showed considerably higher results compared to the glycerin-soaked group. The inferior mechanical properties of both wet groups were attributed to the surface absorption of water and glycerin. A similar trend was observed for samples that underwent artificial aging, although with lower results compared to non-aged groups.

Nowacki et al. [71] conducted a similar study using Anycubic 3D printing UV-sensitive resin and printing via mask stereolithography (mSLA). The specimens were washed in 2-propanol for 10 min before a 30 min post-curing while inside a water-filled glass container. Flexural strength tests showed significant improvement for the wet-cured samples. The UV light passage through the specimens was limited in the wet state due to absorption by the layers of glass and water, which improved the surface hardness while increasing plasticity in the inner parts. Additionally, the hardness increased in the wet state due to oxygen limitation in water, which slowed down polymerization. This study emphasized that the impact of oxygen was more crucial than that of UV light [60]. The oxygen inhibition layer might also influence the shape stability of the 3D-printed products [92,93].

Another study by Vara et al. [46] demonstrated that samples post-cured in a nitrogen-rich atmosphere showed significantly better dimensional accuracy compared to those cured without nitrogen. This suggests that nitrogen post-curing prevents the formation of an oxygen-inhibited layer, leading to improved polymerization and mechanical properties.

Tangpothitham et al. [107] further explored the effects of post-polymerization by autoclaving 3D-printed dental resins. They found that autoclaving at 132 °C for 4 min significantly reduced the elution of residual monomers (UDMA, HEMA, and EGDMA), increased surface microhardness, and maintained the flexural modulus. Despite some linear dimensional changes, the overall fit of the dental appliances remained clinically acceptable. Additionally, autoclaving reduced water sorption and solubility, contributing to improved long-term stability of the 3D-printed dental appliances.

These findings underscore the critical role of optimizing post-curing conditions to enhance the mechanical properties, biocompatibility, and stability of 3D-printed dental materials.

Kirby et al. [96] compared different post-curing units, including less-expensive alternatives to the manufacturer’s recommended unit. The results showed minimal differences in the degree of conversion (DC) among the units, suggesting that alternative curing units might be viable options. However, the study highlighted the importance of curing time over the type of curing unit used. It is worth noting that the study did not explore curing in a nitrogen-rich environment, which might further enhance the DC.

Chang et al. [94] discovered that post-curing 3D-printed parts in various locations within post-polymerization devices affect their color, translucency, microhardness, and flexural strength. Additionally, they found that thermocycling caused changes in color and translucency and diminished the mechanical properties of post-polymerized resins.

### 4.4. Polishing and Surface Treatment

The final phase in the post-processing of 3D-printed objects involves surface polishing and treatment. The necessity for improved surface finishes and enhanced surface roughness is particularly pronounced in additive manufacturing, driven by the specific demands for application-appropriate surface textures and aesthetic considerations [9]. Achieving such refinements often entails the application of various coating techniques to the 3D-printed surfaces, thereby enabling the creation of functionally tailored devices [9]. In the realm of biomedical applications, ensuring biocompatibility is paramount. This is typically addressed through two prevalent methods: firstly, sterilization, a process that eradicates living cells, viable spores, viruses, and viroids from a surface; and secondly, autoclaving, a method of steam sterilization, which is widely recognized for its effectiveness [58].

The selection of an appropriate surface treatment method is critical, as it can significantly impact other material properties. Linares-Alvelais et al. [58] demonstrated that the application of autoclave sterilization on 3D-printed materials could detrimentally affect their tensile properties and lead to surface cracking. In their study, they proposed an alternative approach: hydrostatic high-pressure (HHP) sterilization. This method, involving a system composed of a vessel for applying closure pressure, not only enhanced the biocompatibility of the materials but also improved their elastic modulus and tensile strength [58].

Gonzales et al. [36] conducted a comparative study on the effects of three distinct sterilization protocols on 3D-printed samples. These protocols included (1) exposure to UV light for 30 min within a biosafety cabinet, (2) immersion in 70% ethanol for 10 min, and (3) autoclave sterilization for 20 min at 121 °C. Their findings indicated that autoclaving led to the deformation of the samples, suggesting its unsuitability for these materials. In contrast, neither the ethanol immersion nor the UV light exposure caused any significant damage to the samples. However, the ethanol treatment did introduce minor defects in the sample’s transparency, unlike the UV treatment. Consequently, Gonzales et al. concluded that UV sterilization emerged as the most effective method for enhancing the biocompatibility of 3D-printed materials without compromising their structural integrity [36].

## 5. Problems, Challenges, and Future Directions in Dental 3D Printing Post-Processing

While post-processing is essential for enhancing the mechanical properties, biocompatibility, and overall quality of 3D-printed dental devices, several challenges remain. One of the most significant issues is the inconsistency in post-processing protocols [50], leading to outcome variability. Different resins and 3D printing technologies require specific post-processing conditions, and the absence of universal guidelines makes it difficult for practitioners to achieve consistent results. For instance, the choice of washing solvent, washing duration, and post-curing conditions can significantly impact the final properties of the printed parts [7,66].

Another challenge is the potential for over-curing, which can make the printed parts brittle and weak, thereby compromising their functionality. Over-curing can also lead to shrinkage and dimensional inaccuracies, particularly when higher post-curing temperatures are used [11,79]. This issue is compounded by the anisotropic nature of 3D-printed materials, which can result in uneven mechanical properties and surface characteristics, depending on the orientation of the print layers [85].

Additionally, the influence of environmental factors such as humidity and temperature during the post-processing phase can adversely affect the printed parts. For example, exposure to body temperature and intraoral moisture can alter the mechanical properties and dimensions of dental appliances [11]. Addressing these environmental variables remains a critical challenge in optimizing post-processing protocols.

Moreover, the economic impact of post-processing cannot be overlooked. The labor-intensive nature of many post-processing steps, such as manual cleaning and surface treatment, increases the overall cost of production. Developing automated post-processing systems could mitigate this issue, but such technologies are still in their infancy and require further research and development [63].

In summary, while post-processing is vital for the success of 3D-printed dental devices, several challenges such as the lack of standardized protocols, risks of over-curing, environmental impacts, and economic considerations must be addressed to optimize these techniques for consistent and reliable outcomes.

Future research in post-processing techniques for dental 3D printing should focus on several key areas. First, the development of standardized protocols tailored to different materials and printing technologies is essential. Establishing guidelines for optimal washing solvents, curing durations, and post-curing conditions would significantly enhance the reproducibility and reliability of printed dental devices [2,7].

Additionally, the advancement of automation in post-processing is crucial. Automating labor-intensive steps, such as support removal and surface treatment, could reduce production costs and minimize human error. Research into integrating these automated systems with existing 3D printing workflows is needed to make post-processing more efficient and cost effective [63].

Another promising direction is the exploration of new post-curing technologies that can enhance the mechanical properties and biocompatibility of printed parts without the drawbacks of traditional methods. For instance, developing post-curing systems that utilize controlled environments, such as inert gas atmospheres, could reduce the risk of over-curing and improve the uniformity of polymerization [11,21].

Furthermore, future studies should investigate the long-term effects of post-processing on the durability and stability of dental appliances in clinical settings. Understanding how post-processing impacts the longevity of these devices when exposed to oral environments is critical for ensuring their safety and effectiveness over time [11,79].

Finally, the integration of artificial intelligence (AI) and machine learning (ML) in post-processing presents an exciting frontier. AI-driven algorithms could optimize post-processing parameters in real-time, based on the specific characteristics of each printed part, leading to more personalized and precise outcomes [9,30].

In conclusion, while current post-processing techniques have enabled significant advancements in dental 3D printing, there is considerable potential for further improvement. By addressing the challenges of standardization, automation, advanced curing technologies, and AI integration, the field can move towards more efficient, cost-effective, and reliable post-processing solutions.

## 6. Conclusions

This review highlights the imperative role of selecting appropriate post-processing techniques to achieve desired mechanical properties, biocompatibility, durability, stability, dimensional accuracy, and aesthetic quality in 3D-printed dental devices. These post-processing steps are categorized into four essential phases: support removal, washing, secondary polymerization, and surface treatments. Each phase demands meticulous consideration of various regulatory factors for optimal outcomes. During support removal, the use of proper tools is vital to prevent damage to the sample. In the washing phase, the selection of a suitable solvent, along with the determination of the appropriate duration and method, is critical. For secondary polymerization, it is necessary to establish the optimal temperature, time, wavelength, and conditions. Finally, surface treatments play a key role in enhancing both the aesthetic qualities and biocompatibility for intraoral applications. However, the unique characteristics of each resin type necessitate a tailored approach to post-processing. This review provides a foundational understanding of the post-processing steps, offering valuable insights for future research in this field.

## Figures and Tables

**Table 1:** Influence of post-processing input variables on critical and non-critical attributes.

Input Variables	Critical Attributes	Non-Critical Attributes
**Solvent Selection** [7,9,14,21,36,63,64,65,66,67,68,69]	Mechanical Strength Biocompatibility Dimensional Accuracy Surface Quality	Aesthetic Appeal Surface Roughness Color Stability
**Washing Duration** [7,36,65,69,70,71,72]	Mechanical Strength Biocompatibility Dimensional Accuracy Flexural Strength Surface Quality	Elastic Modulus Water Absorption
**Washing Method** [14,46,67,68,73,74,75]	Mechanical Strength Biocompatibility Dimensional Accuracy Surface Quality	Surface Roughness Water Absorption
**Post-Curing Temperature** [2,9,11,14,21,66,76,77,78,79,80,81,82,83,84]	Mechanical Strength Biocompatibility Dimensional Accuracy Flexural Strength Degree of Conversion Surface Quality	Elastic Modulus Water Absorption
**Post-Curing Duration** [2,14,21,38,41,48,49,66,70,71,79,82,83,84,85,86,87,88]	Mechanical Strength Biocompatibility Degree of Conversion Dimensional Accuracy Flexural Strength Surface Quality	Elastic Modulus Surface Roughness Color Stability
**Post-Curing Wavelength** [16,48,84,88,89]	Mechanical Strength Biocompatibility Degree of Conversion Dimensional Accuracy Surface Quality	Surface Roughness Water Absorption
**Post-Curing Condition** [46,65,71,83,90,91,92,93,94]	Mechanical Strength Biocompatibility Dimensional Accuracy Surface Quality	Surface Roughness Water Absorption

**Table 2:** Summary of selected post-processing studies for 3D-printed dental materials.

No.	Authors	Year	Type of Material and 3D-Printing Technique	Varied Factors	Tested Factors	Post-Processing Results
**1**	Perea-Lowery et al. [2]	2021	IMPRIMO^®^ LC Denture (*DLP*) Palapress^®^ (autopolymerization) Paladon^®^ 65 (heat-polymerization)	Assessing the effect of (1) Two post-curing devices: Imprimo Cure (UV LED, nitrogen gas atmosphere) and Form Cure (LED)Dry post-curing (2) Dry post-curing and post-curing after 30 days of water storage	Flexural strength Elastic modulus Fracture toughness Work of fracture Water sorption Water solubility	Flexural strength and elastic modulus were higher after post-curing but decreased after 30 days in water storage. Water sorption and solubility were better-managed post-curing.
**2**	Uzcategui et al. [14]	2018	PEGDA/PETMP resin and dual-cure approach (*SLA*)	Solvent wash method (rinsing vs. submerging) UV exposure post-curing effect on the modified resin	Shrinkage Plasticization Compressive modulus Degree of conversion	Post-curing improved compressive modulus, while the degree of conversion increased, reducing plasticization.
**3**	Jindal et al. [66]	2020	Dental LT clear resin material (*SLA*)	Time and Temperature of post-curing procedure	Compression strength test	Post-curing at higher temperatures and longer times improved the compression strength significantly.
**4**	Jang et al. [69]	2021	Vericom MAZIC D TEMP dental resin (*DLP*)	Washing time Washing solution	Surface roughness Contact angle Surface energy Residual monomers Degree of conversion Flexural strength	Longer washing times reduced residual monomers, enhancing the degree of conversion and flexural strength.
**5**	Scherer et al. [65]	2022	NextDent C&B MFH N1 (*DLP*)	Post-polymerization condition (dry and wet) Post-polymerization duration Aged and nonaged groups	Flexural strength Fracture resistance	Dry post-polymerization improved flexural strength and fracture resistance compared to wet conditions. Aged groups showed lower performance.
**6**	Snowwhite et al. [63]	2022	Not mentioned	Washing solutions	Flex Strain (Extension) Flexural Modulus Maximum Stress Surface quality	Water-based washing solutions enhanced flexural modulus and maximum stress, while improper solutions reduced surface quality.
**7**	Bardelcik et al. [64]	2021	Polymethyl Methacrylate (PMMA) resin (*SLA*)	Washing solutions	Uniaxial tensile test Stress–Strain	Certain washing solutions improved tensile properties, while others had adverse effects on stress–strain behavior.
**8**	Alsandi et al. [11]	2021	Urethane dimethacrylate (UDMA)-based (*DLP*)	Effects of additional heat to the post-curing process	Ultimate tensile strength Degree of conversion	Additional heat during post-curing significantly improved the tensile strength and degree of conversion.
**9**	Reymus et al. [84]	2019	Experimental resin (*DLP*) NextDent C&B (*DLP*) Freeprint temp (*DLP*) 3Delta temp *(DLP)*	Post-curing Build direction Aging	Fracture load of fixed Dental prostheses (FDPs)	Post-curing and optimal build direction improved fracture load, while aging reduced it.
**10**	Tzeng et al. [38]	2021	Dive urethane acrylates (UAs) (*DLP*)	Post-curing duration	Surface characterization Degree of conversion	Extended post-curing duration led to improved surface characteristics and higher degree of conversion.
**11**	Bagis et al. [82]	1997	Commercial resin composite (Herculite XRV, shade A2, lot 610292, Kerr/Sybron, Orange, CA, USA)	Post-curing time and temperature	Degree of conversion	Higher post-curing time and temperature increased the degree of conversion, improving the final properties of the composite.
**12**	Scherer et al. [65]	2022	NextDent C&B MFH Shade N1; 3DSysytems (*DLP*)	Washing solution Washing duration Aged and nonaged groups	Flexural strength	Increased washing duration and solution concentration improved flexural strength, though aged groups showed lower overall performance.
**13**	Lambart et al. [73]	2022	FREEPRINT^®^ splint 2.0, Detax (*DLP*)	Washing solutions	Roughness Flexural strength Cytotoxicity	Proper washing solutions reduced roughness and enhanced flexural strength while maintaining low cytotoxicity levels.
**14**	Wu et al. [16]	2019	Photocurable ink (*DLP*)	Post-curing UV intensity and thickness of the strip	Distortion and accuracy Degree of conversion	Higher UV intensity during post-curing improved accuracy and degree of conversion but led to increased distortion.
**15**	Mayer et al. [67]	2021	3Delta Etemp (*DLP*) Free-print (*DLP*) Temp PRINT (*DLP*) C&B C&B Micro Filled Hybrid (*DLP*)	Washing solutions Washing method (rinsing, centrifugal force)	Degree of conversion Surface roughness Martens’ hardness Biaxial flexural strength	Centrifugal washing improved the degree of conversion and surface roughness, leading to better mechanical properties.
**16**	Xu et al. [70]	2021	Dental LT Clear Resin V1, Formlabs (*SLA*)	Washing time	Flexural strength Cytotoxicity Surface characterization Water sorption and water solubility	Optimal washing time improved flexural strength and reduced cytotoxicity, with no significant effect on water sorption and solubility.
**17**	Garcia et al. [86]	2020	Formlabs’ Clear resin (*SLA*)	Effect of post-curing time	Final tensile properties	Enhanced washing and extended post-curing improved tensile strength and surface quality while ensuring biocompatibility.
**18**	Monzón et al. [85]	2017	Castable Blend (*DLP*) VisiJet^®^ FTX Green (*DLP*) Industrial Blend (*DLP*)	Effect of post-processing	Eliminating anisotropy	Post-processing reduced anisotropy, improving uniformity and mechanical performance across different orientations.
**19**	Nowacki et al. [71]	2021	Translucent green resin by Anycubic (Shenzhen, China) (*mSLA*)	Washing duration Post-curing duration	Tensile strength Flexural strength Hardness Surface quality/roughness	Extended washing adversely affected tensile strength due to void formation, while flexural strength remained unaffected.
**20**	Katheng et al. [79]	2020	Methacrylate-based photopolymer resin (Clear resin; Formlabs) (*SLA*)	Post-curing duration Post-curing temperature	Dimensional accuracy Degree of polymerization	Higher post-curing temperature led to dimensional distortion, while an optimal temperature of 60 °C maximized polymerization.
**21**	Miedzinska et al. [83]	2020	Formlabs’ Durable Resin (*SLA*)	Effects of post-curing in static, dynamic, and SHPB (the highest strain rate) conditions	Degree of conversion Stress–strain	Higher post-curing temperatures improved mechanical properties in shorter times, with static and dynamic conditions showing significant changes.
**22**	Mayer et al. [68]	2021	Freeprint temp (*DLP*) Temp PRINT (*DLP*) C&B MFH (*DLP*)	Washing solutions Washing method (centrifugal force, immersion)	Fracture load Two-body wear	Centrifugal washing improved fracture load and wear resistance while washing solutions had varying effects on mechanical properties.
**23**	Kim et al. [90]	2020	Methacrylated PDMS-macromer (*DLP*)	Post-curing conditions	Post-curing shrinkage	Optimized post-curing reduced shrinkage, improving dimensional accuracy and overall part integrity.
**24**	Riccio et al. [20]	2021	Formlab resins (*SLA*)	Post-curing effects	Maximum tensile strength Young’s modulus Brittleness	Extended post-curing improved tensile strength and modulus but increased brittleness.
**25**	Cortés et al. [95]	2020	Developed carbon nanotube (CNT) reinforced composites (*DLP*)	Post-curing effects	Young’s modulus Three-point bending strength	Post-curing enhanced Young’s modulus and bending strength, optimizing the mechanical properties of the composites.
**26**	Zachary Zguris [88]	2016	Formlabs resins (*SLA*)	Post-curing wavelength effect	Tensile modulus Tensile strength	Specific wavelengths during post-curing significantly increased tensile modulus and strength.
**27**	Hague et al. [81]	2004	Accura SI40 and SL7560 Resins (*SLA*)	Post-curing levels (UV and thermal)	Tensile properties Flexural properties Impact properties	Higher post-curing levels improved all mechanical properties, with thermal post-curing providing the best results.
**28**	Xu et al. [70]	2021	Dental LT Clear Resin V1, Formlabs (*SLA*)	Post-cure time	Cytotoxicity Surface characterization Roughness measurements Flexural strength	Extended post-cure times improved surface characteristics and flexural strength but did not significantly affect cytotoxicity.
**29**	Oh et al. [72]	2023	Acrylate polymer (*DLP*)	Washing solution temperature (N/T, 30 °C, 40 °C, 50 °C) and washing time (5, 10, 15, 30, 60 min)	Degree of conversion, cell viability, flexural strength, Vickers hardness	Higher washing temperatures increased degree of conversion and cell viability but reduced flexural strength and hardness.
**30**	Kirby et al. [96]	2024	MSLA Dental Modeling Resin (*LCD*)	Post-curing units: Phrozen Cure V2, SUNUV (acrylic nail UV LED), Triad^®^ 2000™ (tungsten halogen light), homemade curing oven. Curing time intervals: 15 min and 45 min	Degree of conversion (DC)	Alternative curing units provided a similar degree of conversion, emphasizing the importance of curing time over unit type.
**31**	Cingesar et al. [97]	2022	Polyacrylate materials (*SLA*)	Post-processing conditions, printing angle, and layer thickness	Mechanical properties (tensile strength, elongation), thermal properties (DSC—glass transition temperature and curing enthalpy), physico-chemical properties (swelling tests, contact angle measurements)	Variations in post-processing conditions and printing angles affected mechanical and thermal properties.
**32**	Vara et al. [46]	2023	DentaGuide (*DLP*) DentaClear (*DLP*)	Post-processing technique: handwashing, sonication, handwashing and sonication mix, nitrogen post-curing	Trueness (median error) Precision (interquartile range) Dimensional accuracy	Nitrogen post-curing significantly improved dimensional accuracy compared to standard post-curing methods.
**33**	Prakash et al. [50]	2024	Various 3D-printed dental resins	Post-processing techniques, material composition	Biocompatibility (toxicity, cell viability) mechanical properties (flexural strength) cytotoxicity	Post-processing optimized biocompatibility and mechanical properties while reducing cytotoxicity.
**34**	Šimunović et al. [61]	2024	Tera Harz TC-85 resin (*DLP*)	Post-curing environments (nitrogen vs. air) Rinsing protocols (centrifuge, ethanol, isopropanol, isopropanol + water)	Degree of conversion (DC) Flexural modulus Hardness	Nitrogen post-curing and specific rinsing protocols enhanced the degree of conversion, flexural modulus, and hardness.
